# Supplementary figures and images for: High pertussis circulation among infants, children and adolescents in Abidjan, Côte d’Ivoire
Source: PLoS One. 2024 Nov 20;19(11):e0310965. doi: 10.1371/journal.pone.0310965 (PMC11578514; doi:10.1371/journal.pone.0310965)

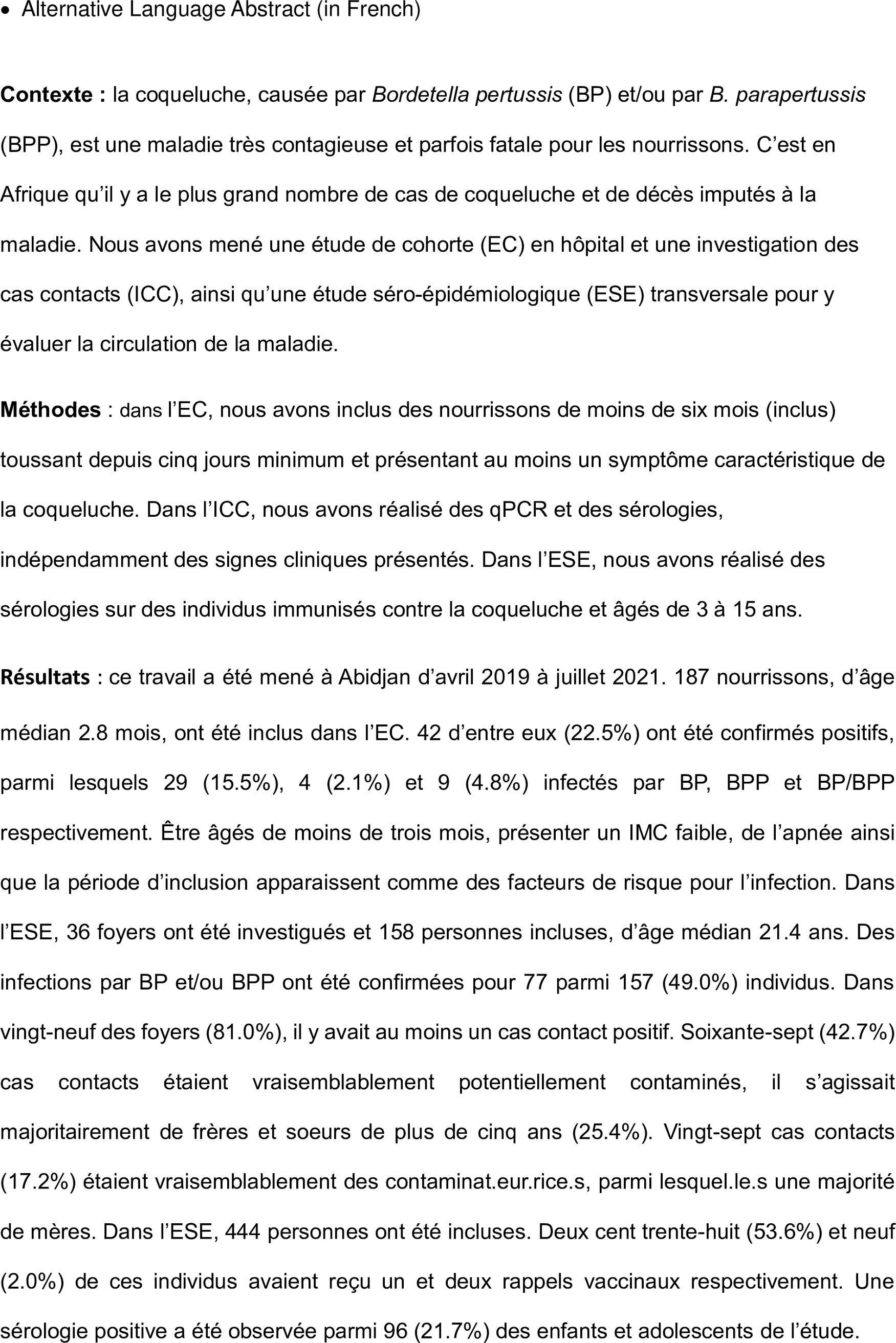

Supplement: S1 File — (TIF) [file pone.0310965.s001.tif]

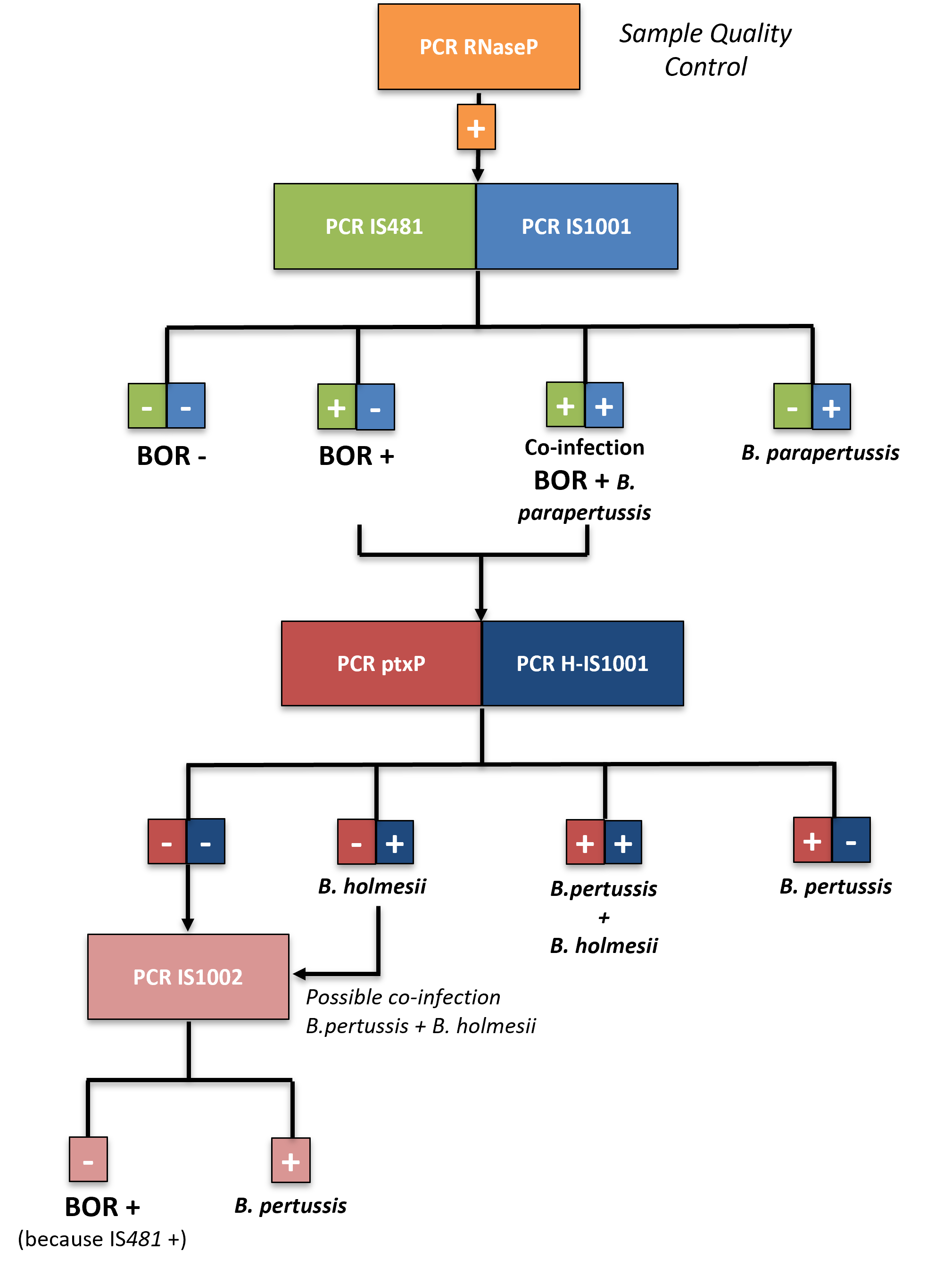

Supplement: S1 Fig — (TIF) [file pone.0310965.s002.tif]

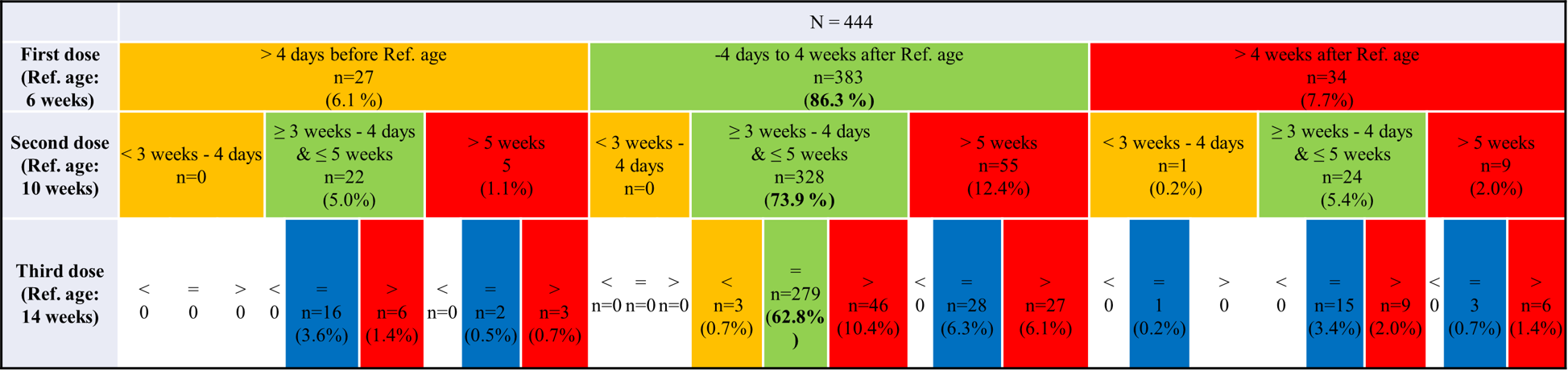

Supplement: S2 Fig — Acceptable age intervals where infants are considered compliant are indicated in the green and blue boxes. The intervals around recommended injection age where the dose is considered as received too early are indicated in yellow boxes, and the intervals where the dose is considered as received too late are indicated in red. (TIF) [file pone.0310965.s003.tif]

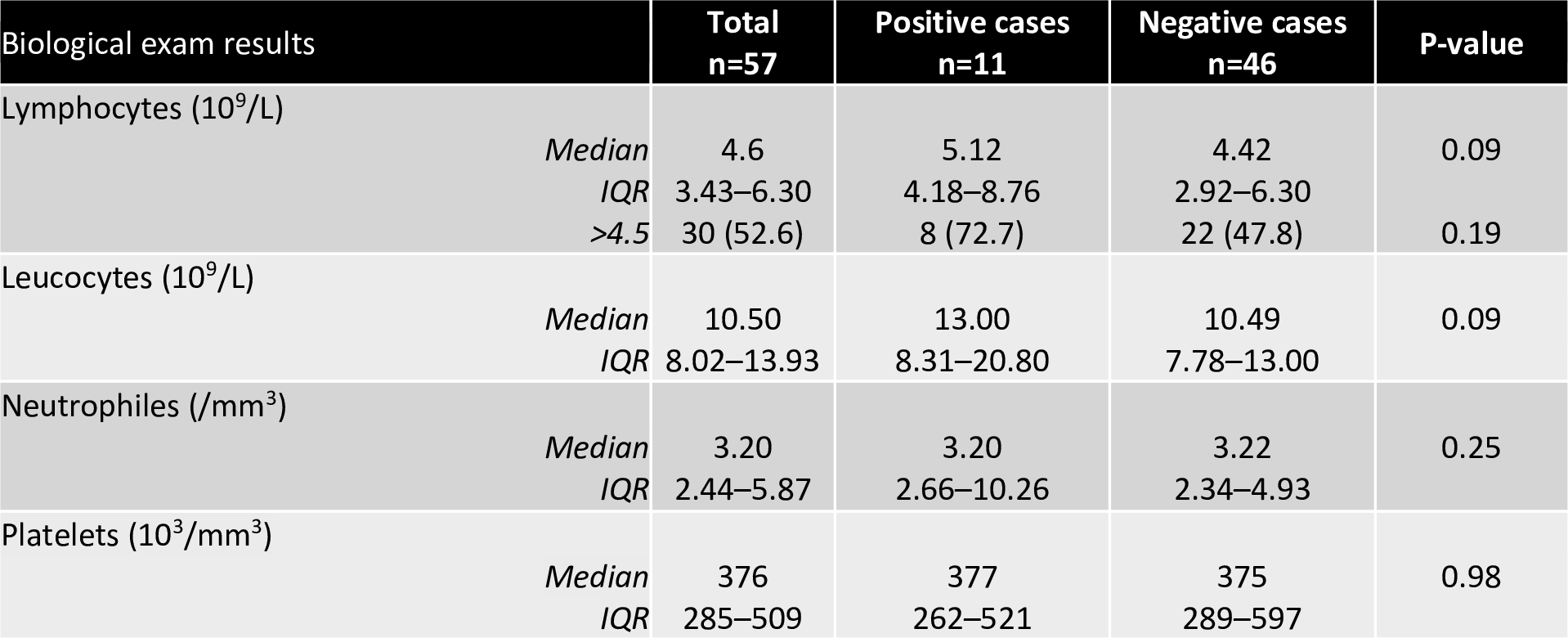

Supplement: S1 Table — (TIF) [file pone.0310965.s004.tif]

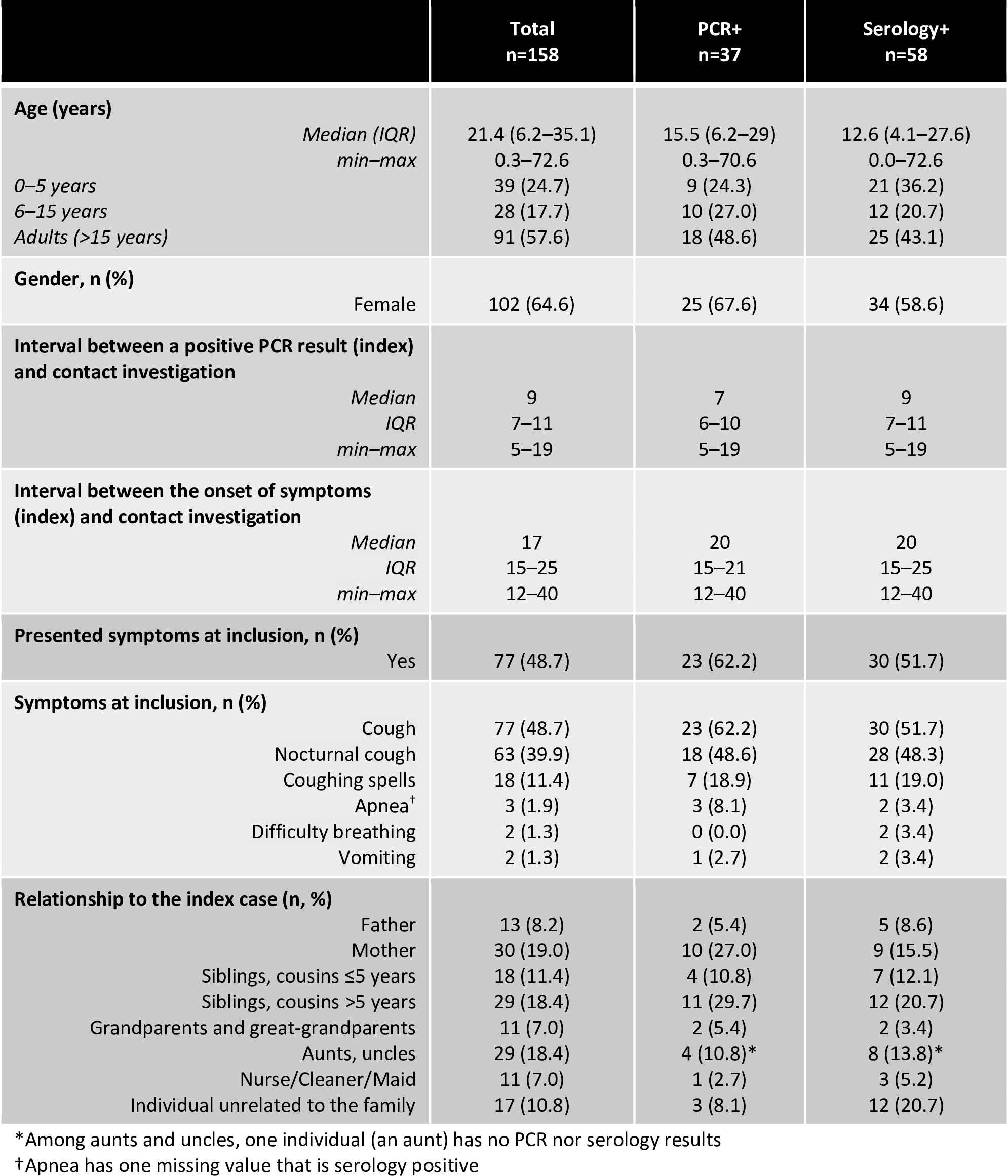

Supplement: S2 Table — (TIF) [file pone.0310965.s005.tif]

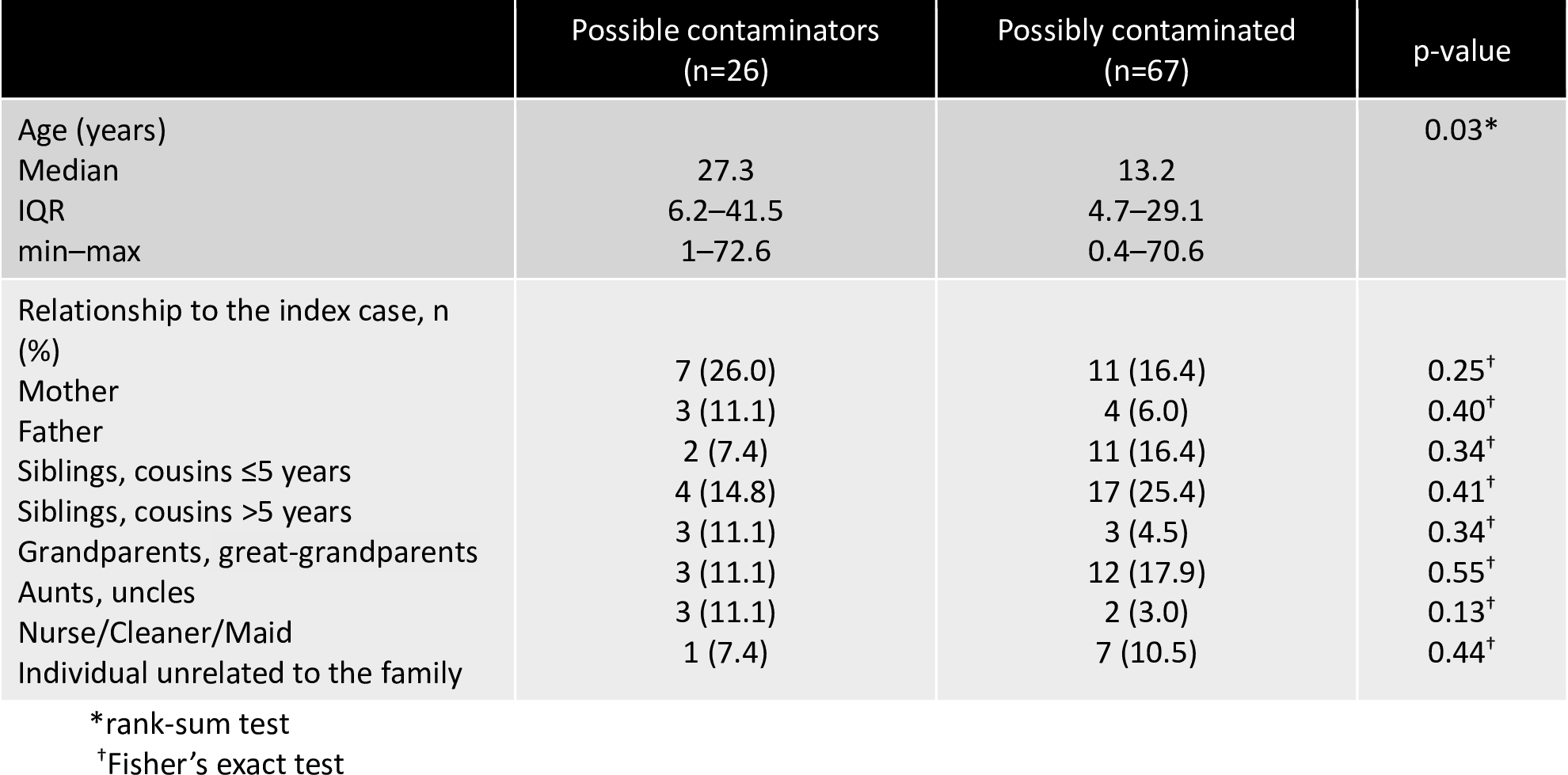

Supplement: S3 Table — n = 157 since missing serology and PCR data for one individual. (TIF) [file pone.0310965.s006.tif]

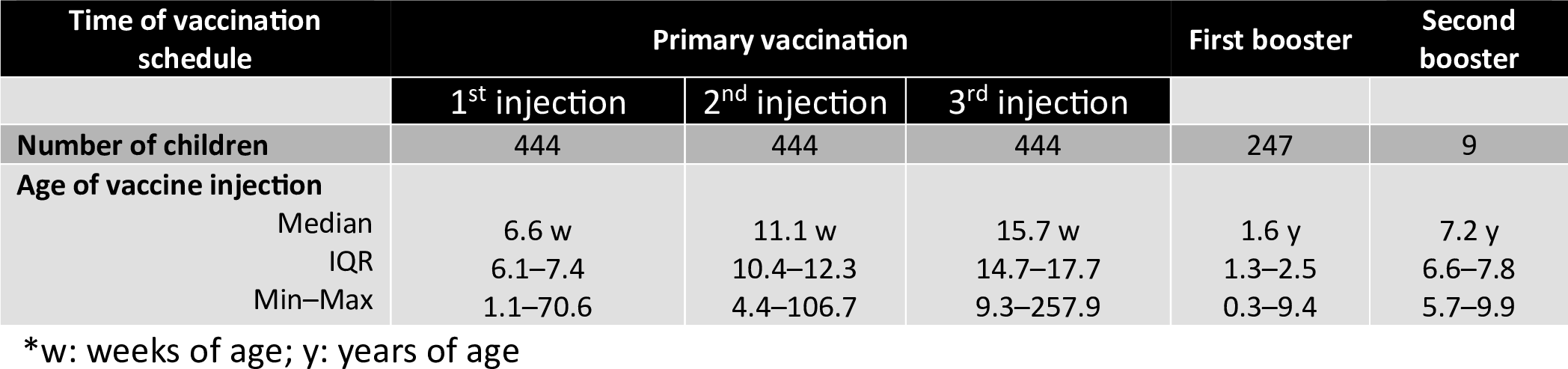

Supplement: S4 Table — (TIF) [file pone.0310965.s007.tif]

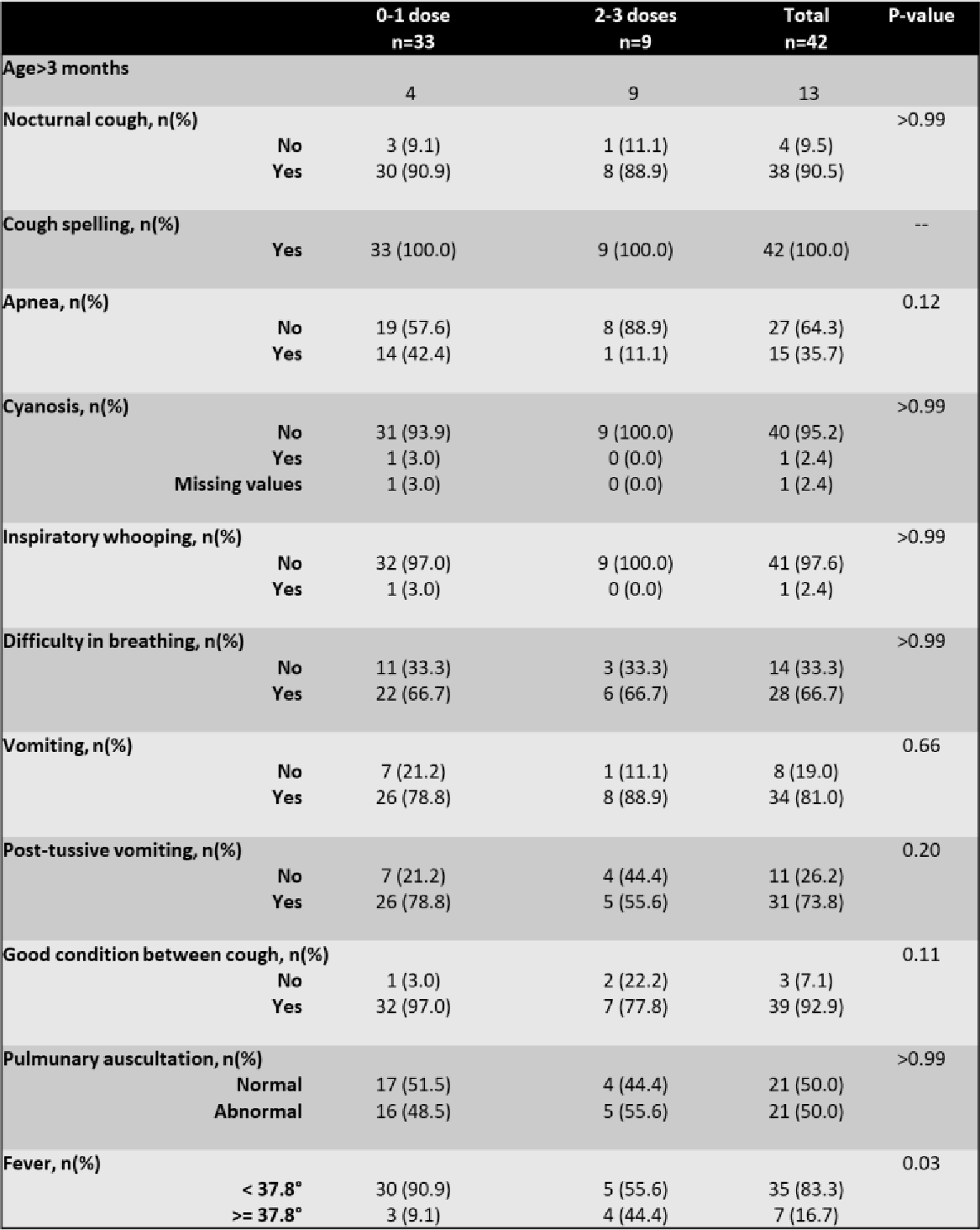

Supplement: S5 Table — (TIF) [file pone.0310965.s008.tif]
