# Supplementary material for: High pertussis circulation among infants, children and adolescents in Abidjan, Côte d’Ivoire
Source: PLoS One. 2024 Nov 20;19(11):e0310965. doi: 10.1371/journal.pone.0310965 (PMC11578514; doi:10.1371/journal.pone.0310965)
Supplement: Attachment — Submitted filename: ResponseToReviewers (1).docx [file pone.0310965.s010.docx]

1) Please add under method that the hospital sample was followed and specify that follow up was to evaluate the course of disease.

*Thank you. We added the sentence ‘for a prospective study in which infants were followed up for an assessment of the evolution of the disease. (Line 127-128).*

2) I suggest that the authors add the Table 2 in the response to supplemental materials and change the “oui” to Yes and the “non” to “No".

*We indeed added the Table after replacement of the ‘Oui’/’Non’, and we mentioned this analysis (Line 336-339), as well as the Table Supplementary 5, whose caption was added at the end of the document (Line 675-676).*

3) Please change limitation to "Limitations and strengths"

*We added it (Line 594).*
